# CryptoType – Public Datasets for MALDI-TOF-MS Based Differentiation of *Cryptococcus neoformans/gattii* Complexes

**DOI:** 10.3389/fcimb.2021.634382

**Published:** 2021-04-19

**Authors:** Mareike Bernhard, Navaporn Worasilchai, Mourine Kangogo, Christine Bii, Wioleta J. Trzaska, Michael Weig, Uwe Groß, Ariya Chindamporn, Oliver Bader

**Affiliations:** ^1^ Institute for Medical Microbiology, University Medical Center Göttingen, Göttingen, Germany; ^2^ Department of Microbiology, Faculty of Medicine, Chulalongkorn University, Bangkok, Thailand; ^3^ Department of Medical Microbiology, Jomo Kenyatta University of Agriculture and Technology, Nairobi, Kenya; ^4^ Center for Microbiology Research, Mycology Laboratory, Kenya Medical Research Institute, Nairobi, Kenya; ^5^ School of Biosciences, Institute of Microbiology and Infection, University of Birmingham, Birmingham, United Kingdom

**Keywords:** MALDI-TOF MS, identification, capsule, *Cryptococcus neoformans* complex, *Cryptococcus gattii* complex

## Abstract

Yeasts of the *Cryptococcus neoformans*/*gattii* species complexes are human pathogens mostly in immune compromised individuals, and can cause infections from dermal lesions to fungal meningitis. Differences in virulence and antifungal drug susceptibility of species in these complexes indicate the value of full differentiation to species level in diagnostic procedures. MALDI-TOF MS has been reported to sufficiently discriminate these species. Here, we sought to re-evaluate sample pre-processing procedures and create a set of publicly available references for use with the MALDI Biotyper system. Peak content using four different pre-processing protocols was assessed, and database entries for 13 reference strains created. These were evaluated against a collection of 153 clinical isolates, typed by conventional means. The use of decapsulating protocols or mechanical disruption did not sufficiently increase the information content to justify the extra hands-on-time. Using the set of 13 reference entries created with the standard formic acid extraction, we were able to correctly classify 143/153 (93.5%) of our test isolates. The majority of the remaining ten isolates still gave correct top matches; only two isolates did not give reproducible identifications. This indicates that the log score cut-off can be lowered also in this context. Ease to identify cryptococcal isolates to the species level is improved by the workflow evaluated here. The database references are freely available from https://github.com/oliverbader/BioTyper-libraries for incorporation into local diagnostic systems.

## Introduction

The group of basidiomycetous yeast of the *Cryptococcus neoformans/gattii* complexes hosts a variety of human pathogenic species, causing infections from skin lesions to fatal meningitis [reviewed in (Kronstad et al., 2011)]. This mainly contributes to morbidity and mortality in patients with underlying immune deficiencies (e.g. HIV), but can also affect immunocompetent hosts. Species of the *C. neoformans/gattii* complexes are readily found in the environment, living, for example, on eucalyptus tree bark, and bird droppings.

The most prominent diagnostic feature of these species are the large capsules of most isolates [reviewed in (O’Meara and Alspaugh, 2012)], which can easily be visualized by, e.g., displacement of India ink stain. India ink does not penetrate the capsule and thus creates a halo around the cells visible in microscopy. The polysaccharides shed from the cell also give rise to efficient and specific serologic tests of cryptococcal infections through serum detection of galactomannan.

Species in this complex have traditionally been divided into four serotypes based on antigenicity of the capsule, forming three varieties: *C. neoformans* var. *grubii* (serotype A), var. *gattii* (serotypes B and C), and var. *neoformans* (serotype D). They are also able to form inter-species hybrids leading to, e.g., an AD serotype (Boekhout et al., 2001). Several genetic methods are available to stratify the different serotypes into further molecular types (Meyer et al., 2003) and characterize hybrid strains. Recently, it has been proposed to formally raise the non-hybrid molecular types to species level (Kwon-Chung et al., 2002; Hagen et al., 2015) and a fifth *C. gattii* lineage has recently been described (Farrer et al., 2019) from environmental and animal specimen.

In clinical samples from Europe most frequently serotype A is found, mainly from immunocompromised patients, e.g. those suffering from AIDS (Kronstad et al., 2011). Highly virulent isolates usually stem from the *C. gattii* complex, which also readily infect immuno-competent hosts. Differences in mean antifungal susceptibility between closely related molecular types have been reported (Trilles et al., 2012; Cogliati et al., 2018; Lee et al., 2019) and *in vitro* differences in cytokine responses (Herkert et al., 2018). Some molecular types, mainly VGII and VGIII, are more prone to be involved in outbreak scenarios (Kidd et al., 2004; Carriconde et al., 2011; Springer et al., 2014). A major difference between *C. neoformans* and *C. gattii* groups is the lack of growth inside macrophages among *C. gattii* isolates, with the notable exception of such outbreak lineages (Voelz et al., 2014).

Together this underlines the benefit of methods easily discriminating between the major molecular types, not only in clinical contexts, but also for epidemiological studies which so far rely on laborious genetic typing [e.g. our own work (Tangwattanachuleeporn et al., 2013; Kangogo et al., 2015; Worasilchai et al., 2017) or others (Fang et al., 2020; Jin et al., 2020)]. MALDI-TOF MS has been established over the past years as a widely used clinical species identification tool and has been shown to be able to discriminate between the seven known molecular types within the *C. neoformans/gattii* complexes (McTaggart et al., 2011; Firacative et al., 2012; Posteraro et al., 2012; Hagen et al., 2015). For *C. gatti* and *C. deuterogatii* differential mass peaks have been described (Jin et al., 2020).

However, this has not been implemented in diagnostic systems, which remain at the point where only *C. neoformans* var. *neoformans/grubii* vs. *C. gattii* complexes can be identified. In part, this may be due to the observation that false species designations above the significance threshold can be observed (Posteraro et al., 2012), and reflect the complexity introduced by hybrid formation between the different linages.

In this study, we created a publicly available MALDI Biotyper database reference (“main spectrum projections”, MSPs) set from 13 type strains of seven recognized non-hybrid subtypes in the *Cryptococcus neoformans*/*gattii* complexes. Their performance using different preprocessing protocols is evaluated on a set of characterized isolates.

## Materials and Methods

### Yeast Strains and Culture Conditions, Chemicals

For long-term storage, *Cryptococcus* isolates were kept at -70°C in cryobank stocks (Mast Diagnostica, Reinfeld, Germany). After thawing, strains were propagated on Sabouraud’s (SAB) agar slants supplemented with 0.5% peptone (casein), 0.5% peptone (meat), and 2% glucose. Before sample preparation, strains were cultivated on SAB agar overnight at 30°C.

For the purpose of text clarity, only the species nomenclature according to Hagen et al. (Hagen et al., 2015) is adopted from here. As references, thirteen strains of the CBS collection (Westerdijk Fungal Biodiversity Institute) were used: three *C. neoformans* (CBS 8710 (molecular type VNI), CBS 10084 (VNII), CBS 10085(VNI)), two *C. deneoformans* (CBS 6900 and CBS 10079 (VNIV)), two *C. gattii* (CBS 6289,and CBS 10078, VGI), two *C. deuterogattii* (CBS 10082, and CBS 10514, VGII) two *C. bacillisporus* (CBS 6955 and CBS 10081, VGIII), one *C. tetragattii* (CBS 10101, VGIV), and *C. decagattii* (CBS 11687, VGIV).

A test set of 153 isolates was assembled from previously characterized collections. This included all Thai strains from Worasilchai et al. (2017), augmented with rare species isolates from Kenya (Kangogo et al., 2015) and the Birmingham laboratory collection, which include strain from various studies [e.g. (Voelz et al., 2014)]. All isolates were typed either previously (Kangogo et al., 2015; Worasilchai et al., 2017) or specifically for the purpose of this study using the *URA5*-RFLP method. The final set contained n=96 *C. neoformans*, n=6 *C. deneoformans*, n=5 *C. gattii*, n=18 *C. bacillisporus*, n=20 *C. deuterogattii*, and n=8 *C. tetragattii* isolates. A negative control group was assembled from mass spectra randomly chosen from those obtained during bacterial (n=86) of fungal (n=403) routine diagnostics.

### *URA5*-RFLP

Restriction fragment length polymorphisms were performed as described previously (Kangogo et al., 2015; Worasilchai et al., 2017). Briefly, genomic DNA was extracted from cells using phenol/chloroform and the *URA5* gene was amplified using URA5 forward (5-ATGTCCTCCCAAGCCCTCGACTCCG-3) and SJ01 reverse (5-TTAAGACCTCTGAACACCGTACTC-3) primers (Meyer et al., 2003). The amplicons obtained were either simultaneously digested with HhaI (20 U/μl) and Sau96I (10 U/μl) or StuI (10 U/µl) alone for 8 hours (all from New England Biolabs). The digestion products were purified using a PCR clean-up kit (NucleoSpin, Macherey-Nagel, Düren, Germany) and visualized on a 3% agarose gel.

### MALDI-TOF MS Preprocessing Protocols

For regular harvest and formic acid-extraction [preprocessing protocol (A) (Bader, 2017)], cells were taken from agar plates by scraping approximately a 1µl loop full of cells and re-suspending them in 300 µl water. 700 µl absolute ethanol was added to a final concentration of 70% (v/v) and vortexes. Cells were spun down at 8500x*g* for 5 min, the supernatant completely discarded and the cells lysed first with 50 µl 70% (v/v) formic acid, and 50 µl pure acetonitrile. Modifications to this protocol tested were for preprocessing protocol (B) that cells were collected in 300 µl 5% (v/v) DMSO_ad_, for preprocessing protocol (C) that DMSO was included in the 70% ethanol washing step to a final volume of 5% (v/v), and for preprocessing protocol (D) that cells were collected in 300 µl water already including an equivalent of ~100 µl glass beads (0.5 mm diameter, Roth, Karlsruhe, Germany). Here, cells were mechanically disrupted in a FP120 fast prep machine (Bio101, Thermo Savant) at setting 4, for 30 sec during the formic acid step.

### Generation of MALDI Biotyper Database References

MSP references for the MALDI Biotyper were generated according to the manufacturer’s guidelines (Kostrzewa and Maier, 2017), using preprocessing protocol A. Spectra from 24 individual spots were gathered on a freshly calibrated (BTS reference standard) Autoflex III system (Bruker Daltonics, Bremen, Germany) using the automated acquisition mode of the Biotyper 3.1. Spectra were processed using the inbuilt MSP generation method, using the standard parameters.

## Results and Discussion

### Method Optimization

**Figure 1:**
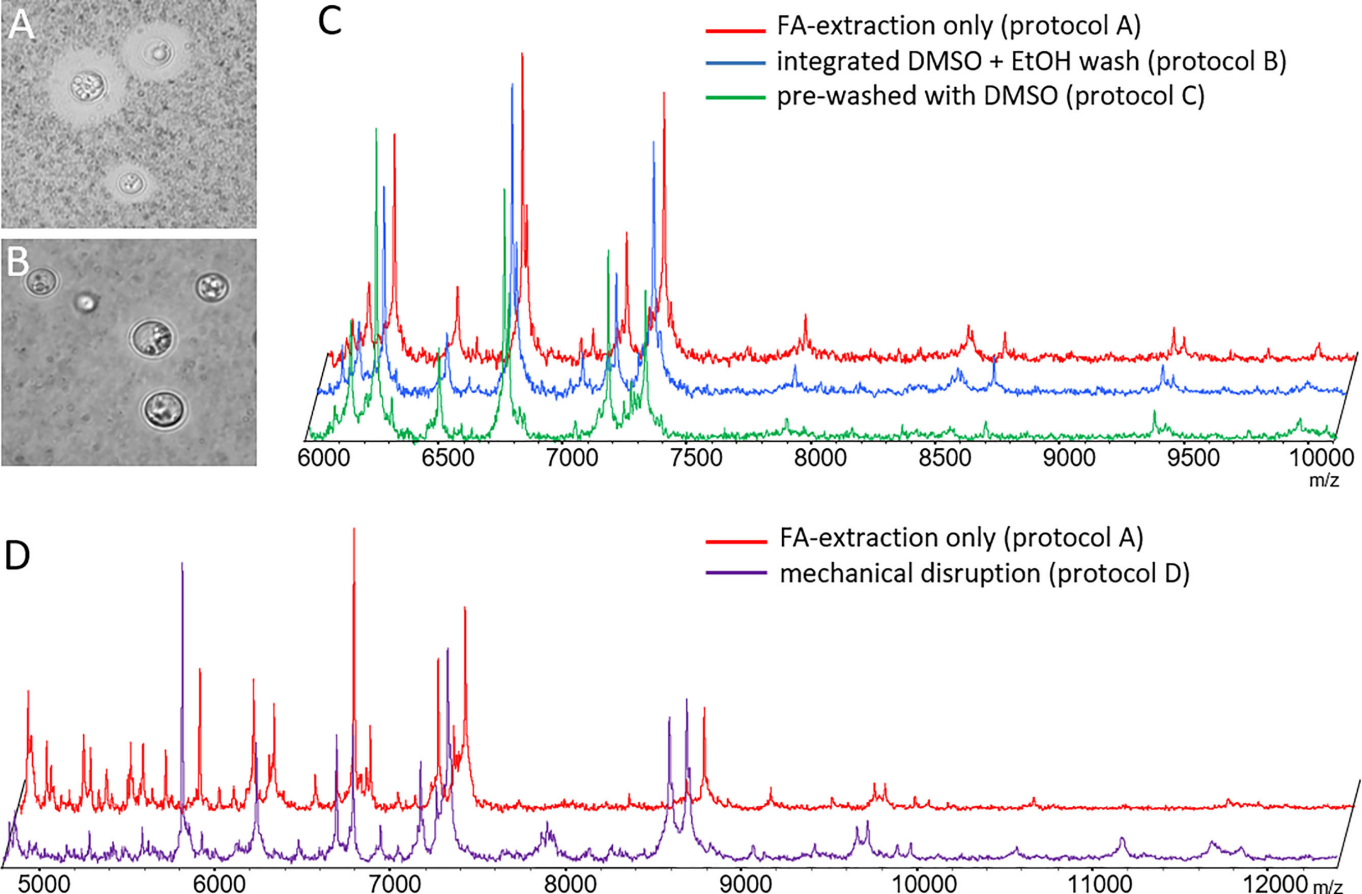
Optimization of sample pre-processing procedures. Phase contrast microscopy of ink-stained cryptococcal cells **(A)** without and **(B)** with DMSO treatment depleting capsular material (protocol B). **(C)** Spectra obtained from de-capsulated cells by protocols B and C were not different from those generated by formic acid extraction alone. **(D)** Spectra obtained from mechanically disrupted cells (protocol D) had similar masses, but differed in relative signal intensity for some, as compared to those obtained by formic acid extraction. Example results shown here for CBS 10485 are valid for all isolates. Signal intensities on y-axes in panels **(C, D)** mainly rely on number of spectra gathered in sum buffer. Spectra have been manually re-scaled on the y-axis for better visual comparison between different experimental conditions, and scaling has been omitted to reflect this fact.

The literature reports that both, removal of cryptococcal capsule can (Thomaz et al., 2016) or does not (Hagen et al., 2015) positively influence spectrum quality. Since the capsule material is soluble in DMSO, we devised pre-processing protocols that would deplete the capsule prior to the regular formic acid/acetonitrile extraction protocol. Both pre-processing protocols, B (**Figures 1A, B**) and C (not shown), efficiently removed capsules in all strains. However, subsequent measurement of mass spectra did not reveal any additional mass signals, or major differences in spectrum quality (**Figure 1C**).

Next, we tested if mechanical disruption of the cells yielded more informative spectra using mechanical disruption (preprocessing protocol D). Indeed, mass spectra recorded from mechanically disrupted cells resulted in more evenly distributed peak intensities across the major mass signals. However, no additional mass signals of high intensity were found (**Figure 1D**).

In our hands removal of the capsule did not result in spectra with higher information content, at any time. Mechanical disruption did reveal some additional masses, but in favor of the lower hands-on-time the original pre-processing protocol A was subsequently used for MSP creation and testing.

### Creation of Single Species MSPs

**Figure 2:**
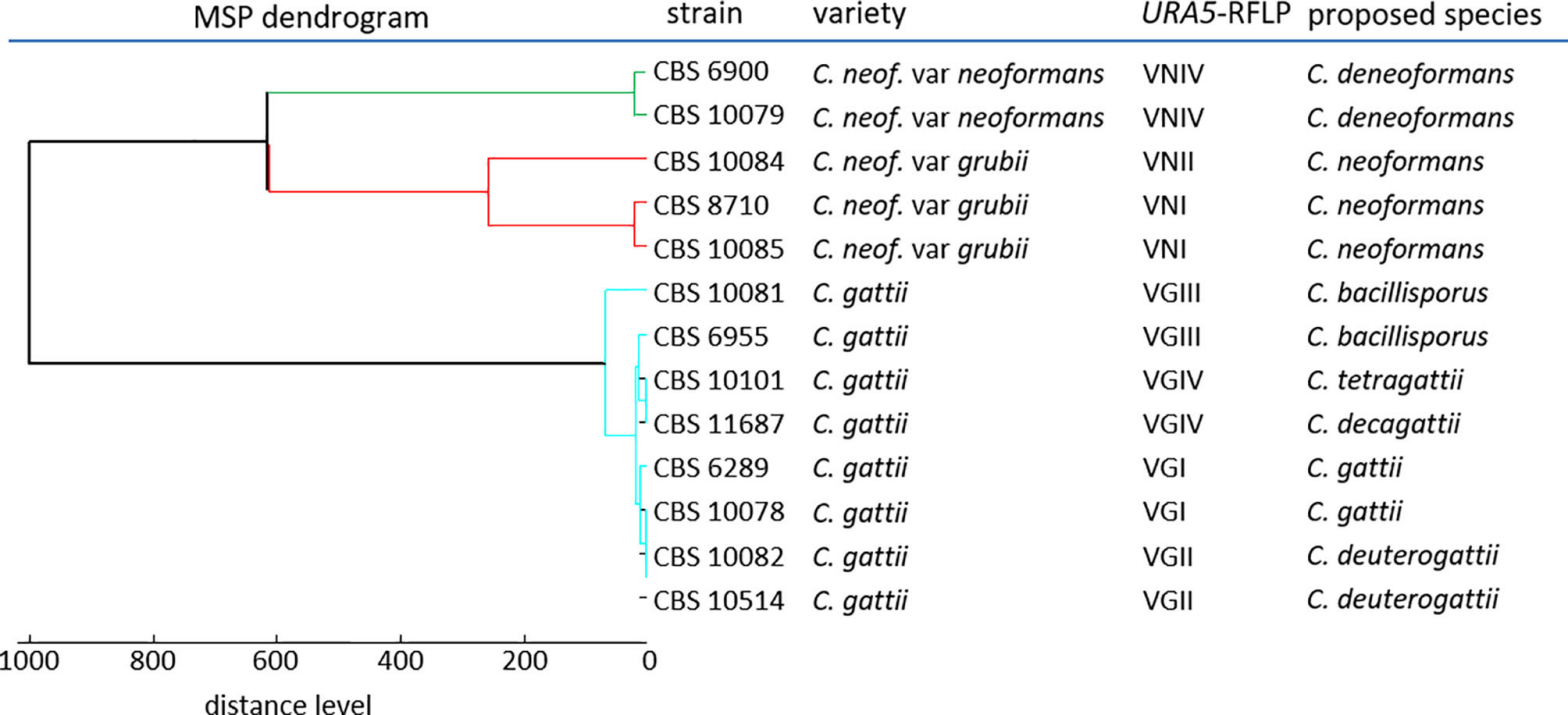
Dendrogram reference MSPs.

Next, we created MSPs for 13 reference strains encompassing seven molecular types of the *C. neoformans/gattii* complexes (Meyer et al., 2003; Hagen et al., 2015), using the standard extraction procedure (pre-processing protocol A). Cluster analysis of the MSPs generated suggested sufficient distance to clearly distinguish between *C. neoformans* complex molecular types VNIV (*C. deneoformans*) and VNI/II, and possibly also between VNI and VNII themselves, but less so among molecular types within the *C. gattii* complex (**Figure 2**).

### Identification Performance

**Figure 3:**
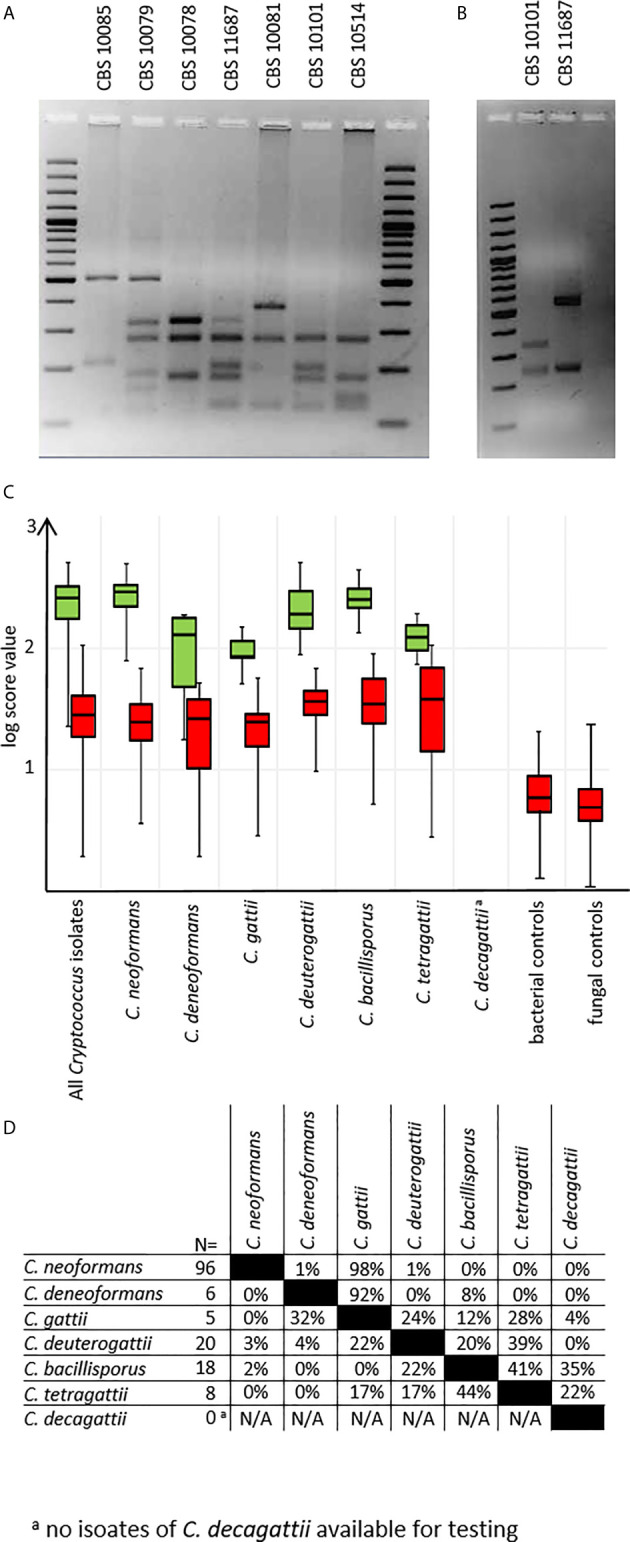
*URA5*-RFLPs. **(A)** *URA5*-RFLP patterns of control isolates. **(B)** CBS10101 (*C. tetragattii*) and CBS 11687 (*C. decagattii*) can be differentiated by digesting the *URA5*-amplicon with StuI. **(C)** log-score values stratified by species/*URA5*-RFLP and matching type. Green: scores of correct matches, red: highest scoring incorrect matches. **(D)** Pairwise percentages of highest scoring *in*correct matches. ^a^ *C*. *decagattii* was not present among testing isolates, as the only strain available for this study was already used to create the MSP reference.

Mass spectra for all test isolates were obtained using preprocessing protocol A. Were MALDI-TOF results using the new MSP set deviated from previous data, *URA5*-RFLP typing was repeated as the gold standard (**Figure 3A**). All but two deviations could be resolved (see below). To discriminate between *C. tetragattii* and potential *C. decagattii* strains, we sequenced the *URA5*-amplicon obtained from CBS 11687 (*C. decagattii*, deposited at Genebank under the accession number MH605184) and compared it to the respective sequence of CBS 10101 (*C. tetragattii*, gene bank accession AY973155). Restriction with StuI was found, and experimentally confirmed, to discriminate the two species (**Figure 3B**). However, there were no further *C. decagattii* isolates among our strains. *C. decagattii* remains a rare species, and only a single isolate of this molecular type (CBS 11687) was available for this study, which was already included in the reference set. Therefore, the final test collection encompassed only six of the seven species used for generation of references.

From the test collection, we were able to correctly identify 143/153 (93.5%) of the isolates on species-level using duplicate spots, with the top log score ≥ 2.000 (**Figure 3C**), as recommended by the manufacturer. Of the remaining ten isolates, eight still gave correct species matches at scores between 1.700 and 1.999, considered only genus-level by the manufacturer. Among the negative control set, there were no results higher than a log score of 1.300, indicating no false positives are to be expected under routine diagnostic conditions (**Figure 3C**). Inconsistent identifications were only observed for two *C. tetragattii* isolates where repetitively top matches of different spots of the same preparation were *C. tetragattii, C. gattii*, or *C. deuterogattii*, all at values above 1.999.

Because of this, and the close relations found during cluster analysis (**Figure 2**), we also inspected the log score difference from the correct to the highest scoring false match for each spot (**Figure 3D**) for those tests where a second species matched above the significance threshold. Only 3% of all tested spots (14 out of 428) matched a second MSP with a log score >1.999. As expected from the cluster analysis, these “best false” second matches were found only among species in the *C. gattii* complex. This was the case for three *C. bacillisporus* isolates giving a second best match with *C. decagattii*, with a log score difference between 0.1 to 0.4. In addition to the two inconsistent *C. tetragattii* isolates discussed above, one additional *C. tetragattii* isolate also gave a second best match with *C. decagattii*. The score values for both matches were near 2.000. The close relationships of the different species will likely also have implications on properly identifying hybrid isolates.

## Conclusion

Cryptococcal typing and species identification is complicated by the ongoing discovery of new species (Farrer et al., 2019), and the formation of inter-species hybrids (Hagen et al., 2015). Nevertheless, our data confirms that proper routine identification of clinically relevant non-hybrid *C. neoformans/gattii* complex molecular types using MALDI-TOF is possible with the current algorithms and standard workflows. In our hands, the only exception was distinguishing the rarer types *C. tetragattii* and *C. decagattii*, which was not sufficiently possible. This may be due to the fact, that only low numbers of isolates of these linages were available for testing.

The MSP sets generated in this study are freely available from https://github.com/oliverbader/BioTyper-libraries for use with the molecular type- (Meyer et al., 2003) or the species nomenclatures (Hagen et al., 2015).

## Data Availability Statement

The datasets presented in this study can be found in online repositories. The names of the repository/repositories and accession number(s) can be found in the article.

## Author Contributions

Performed experiments: MB, NW, MK, OB. Contributed typed strains: NW, MK, CB, WT, AC. Wrote the manuscript: MB, AC, OB. Prepared the revision: MB, OB. Supervised the study: MW, UG, AC, OB. All authors contributed to the article and approved the submitted version.

## Funding

This study received funding from Thai-German mobility scheme “CryptoType” to AC and OB (grant number 01DP13001). Article publishing fees were covered by the Open-Access-publications funds of the Universitätsmedizin Göttingen.

## Conflict of Interest

The authors declare that the research was conducted in the absence of any commercial or financial relationships that could be construed as a potential conflict of interest.
